# Supplementary material for: PermQRDroid: Android malware detection with novel attention layered mini-ResNet architecture over effective permission information image
Source: PeerJ Comput Sci. 2024 Oct 17;10:e2362. doi: 10.7717/peerj-cs.2362 (PMC11623236; doi:10.7717/peerj-cs.2362)
Supplement: Supplemental Information 6 [file peerj-cs-10-2362-s006.docx]

| Author | Year | Dataset | Number of APK | Analysis Type | Feature | Feature Selection | Classifier |
| --- | --- | --- | --- | --- | --- | --- | --- |
| Yadav et al. | 2022 | R2-D2 | 5986 | Static | Dex file byte image | --- | EfficientNet-B4 |
| Yen & Sun | 2020 | --- | 1440 | Static | Tf-idf to image | --- | CNN |
| Xiao & Yang | 2020 | Google Play Store, AMD | 10540 | Static | Dalvik byte code image | --- | CNN |
| Zhu et al. | 2023 | Google Play Store, Virusshare | 3187 | Static | Permission, Hardware and API calls image |  | MSerNetDroid (CNN) |
| Aurangzeb et al. | 2024 | Drebin ,Kronodroid, Androzoo | 24746 | Static | Dex file byte image | PCA | XGBoost |
| Taşyürek & Arslan | 2023 | Drebin, Genome, Arslan (Google Play Store) | 7721 | Static | Permission RGB image |  | YoloV5 |
| Arslan & Taşyürek | 2022 | Drebin ,Genome, VirusTotal | 1920 | Static | Permission 2D-code image |  | CNN |
