# Supplementary material for: PermQRDroid: Android malware detection with novel attention layered mini-ResNet architecture over effective permission information image
Source: PeerJ Comput Sci. 2024 Oct 17;10:e2362. doi: 10.7717/peerj-cs.2362 (PMC11623236; doi:10.7717/peerj-cs.2362)
Supplement: Supplemental Information 7 [file peerj-cs-10-2362-s007.docx]

|  | Class | Number of Apps | Source |
| --- | --- | --- | --- |
| Dataset1 | Benign | 20.000 | Androzoo |
|  | Malware | 20.000 | Androzoo |
| Dataset2 | Benign | 961 | Google Play Store |
|  | Malware | 6661 | Drebin,Genome |
| Dataset3 | Benign | 1000 | APKPure |
|  | Malware | 1000 | Drebin |
| Dataset4 | Benign | 250 | Google Play Store |
|  | Malware | 250 | CICMalDroid |
