# Supplementary material for: PermQRDroid: Android malware detection with novel attention layered mini-ResNet architecture over effective permission information image
Source: PeerJ Comput Sci. 2024 Oct 17;10:e2362. doi: 10.7717/peerj-cs.2362 (PMC11623236; doi:10.7717/peerj-cs.2362)
Supplement: Supplemental Information 8 [file peerj-cs-10-2362-s008.docx]

| Architecture | Input Size | Parameter | Size (MB) |
| --- | --- | --- | --- |
| Xception | 299x299x3 | 22.9 M | 88 |
| VGG-16 | 224x224x3 | 138.4 M | 528 |
| ResNet50 | 224x224x3 | 25.6 M | 98 |
| ResNet101 | 224x224x3 | 44.7 M | 171 |
| Inception v3 | 299x299x3 | 23.9 M | 92 |
| MobileNet | 224x224x3 | 4.3 M | 16 |
| DenseNet | 224x224x3 | 8.1 M | 33 |
| Proposed Architecture | 10x10x1 | 399.618 K | 4.94 |
