# Supplementary figures and images for: PermQRDroid: Android malware detection with novel attention layered mini-ResNet architecture over effective permission information image
Source: PeerJ Comput Sci. 2024 Oct 17;10:e2362. doi: 10.7717/peerj-cs.2362 (PMC11623236; doi:10.7717/peerj-cs.2362)

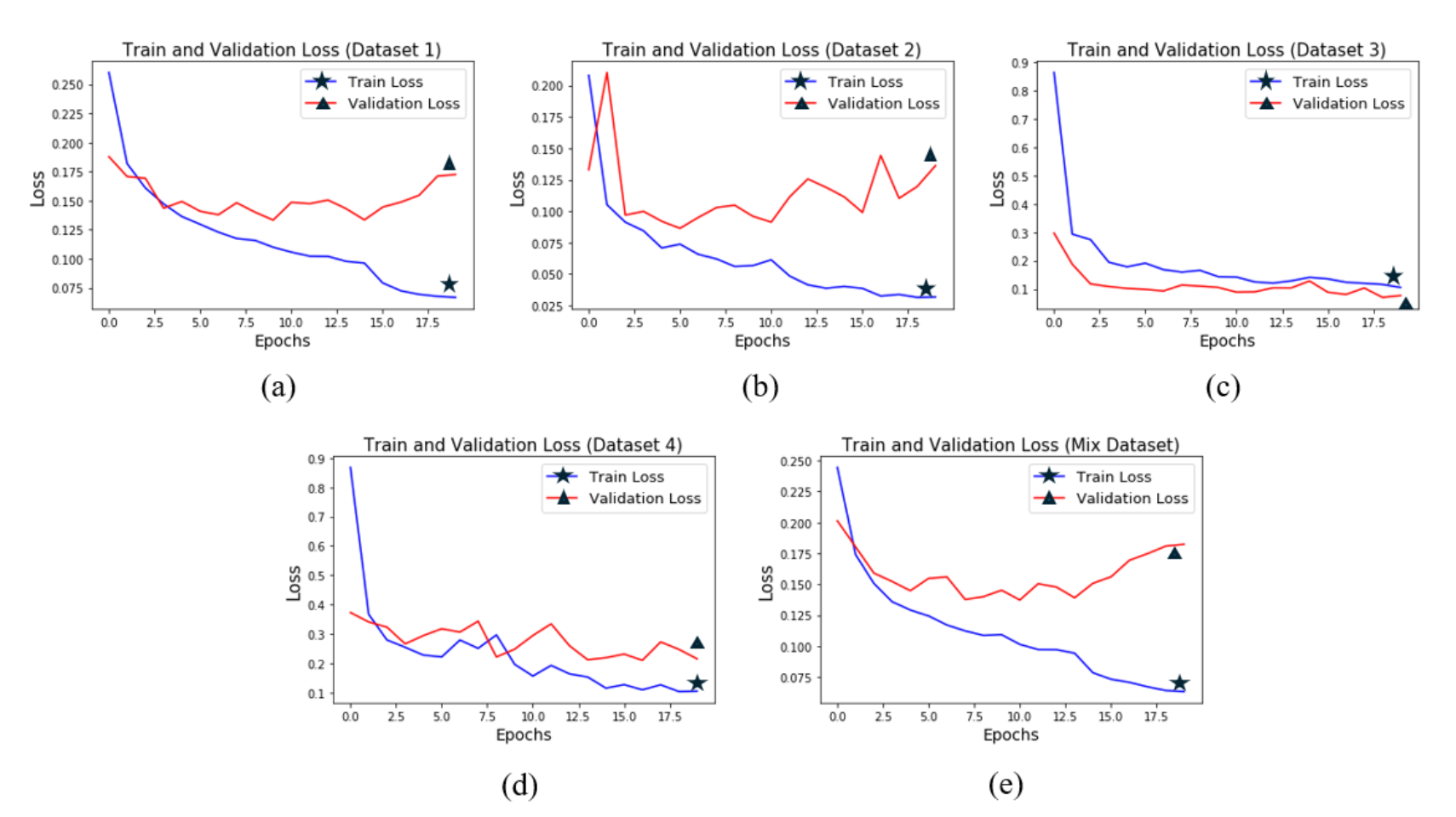

Supplement: Supplemental Information 9 [file peerj-cs-10-2362-s009.png]

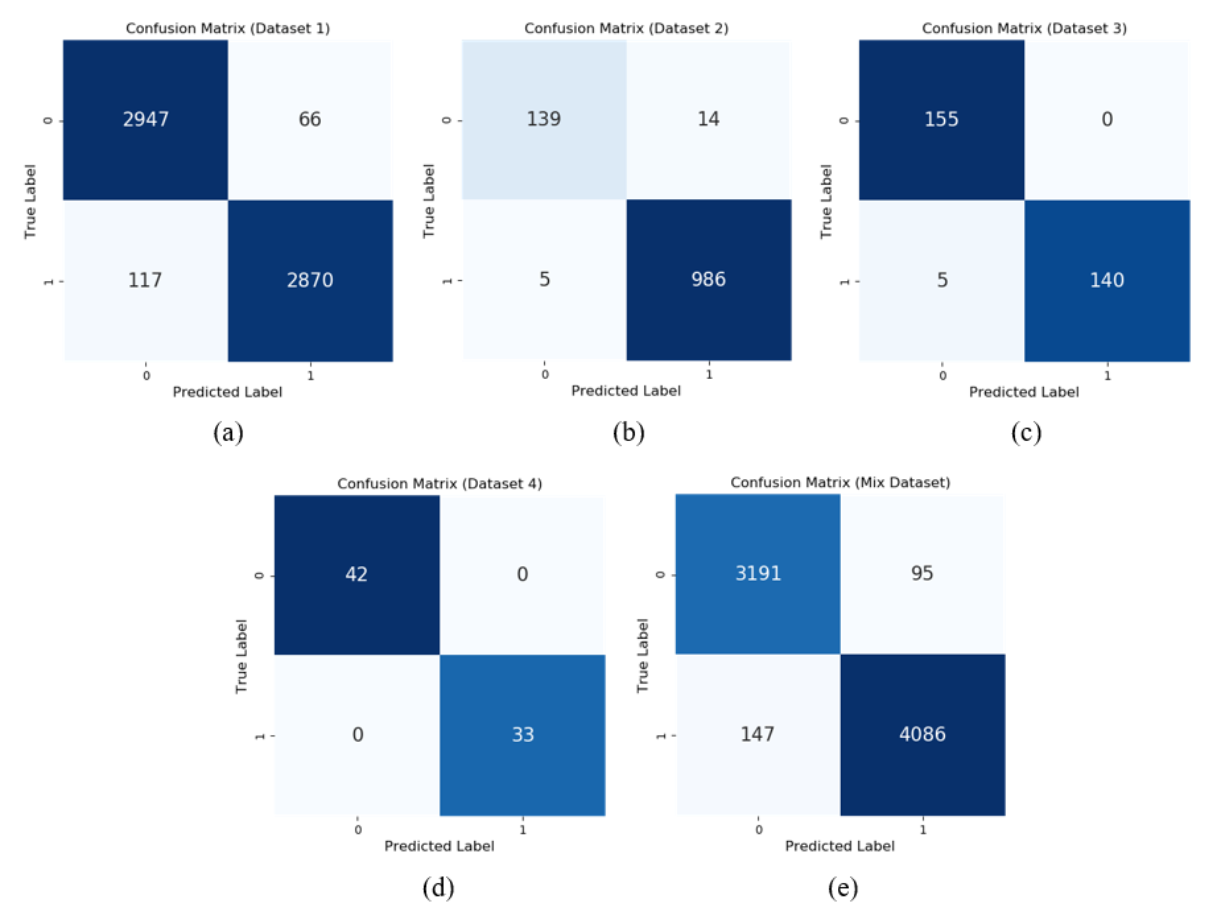

Supplement: Supplemental Information 10 [file peerj-cs-10-2362-s010.png]

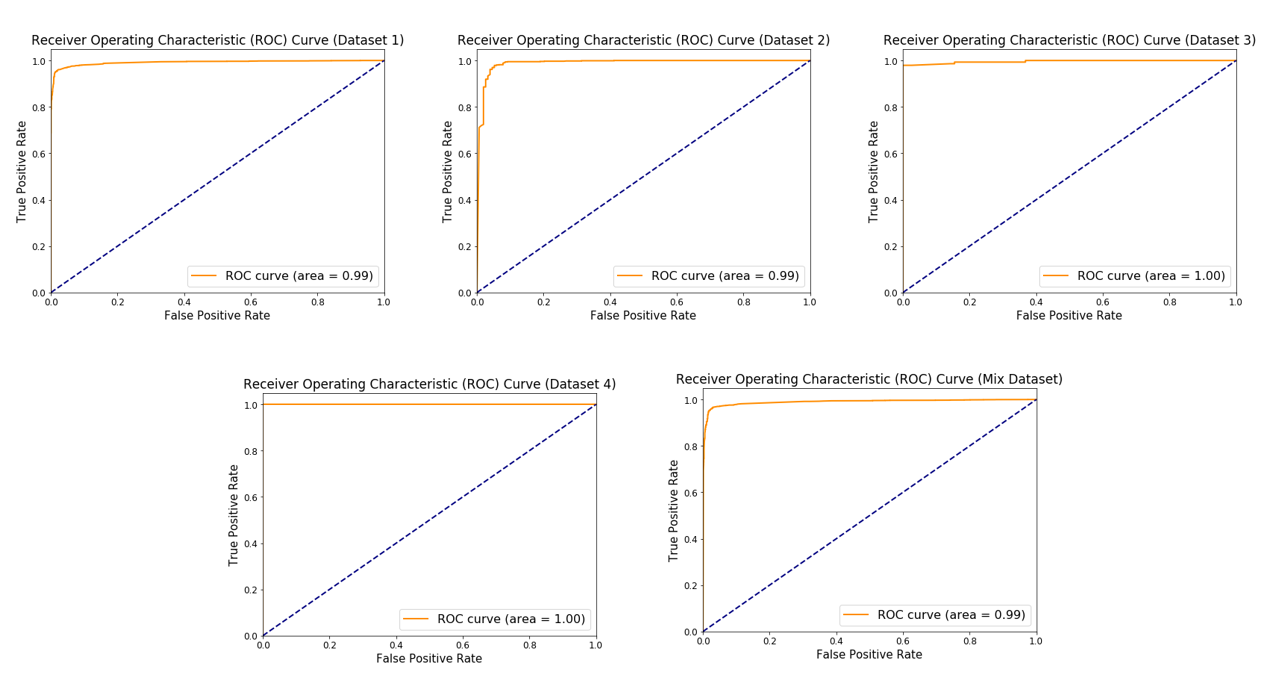

Supplement: Supplemental Information 11 [file peerj-cs-10-2362-s011.png]
